# Supplementary material for: Wet-Spun Polycaprolactone Scaffolds Provide Customizable Anisotropic Viscoelastic Mechanics for Engineered Cardiac Tissues
Source: Polymers (Basel). 2022 Oct 28;14(21):4571. doi: 10.3390/polym14214571 (PMC9657938; doi:10.3390/polym14214571)
Supplement: Supplementary file 1 [file polymers-14-04571-s001.zip › polymers-1972056-supplementary.pdf]

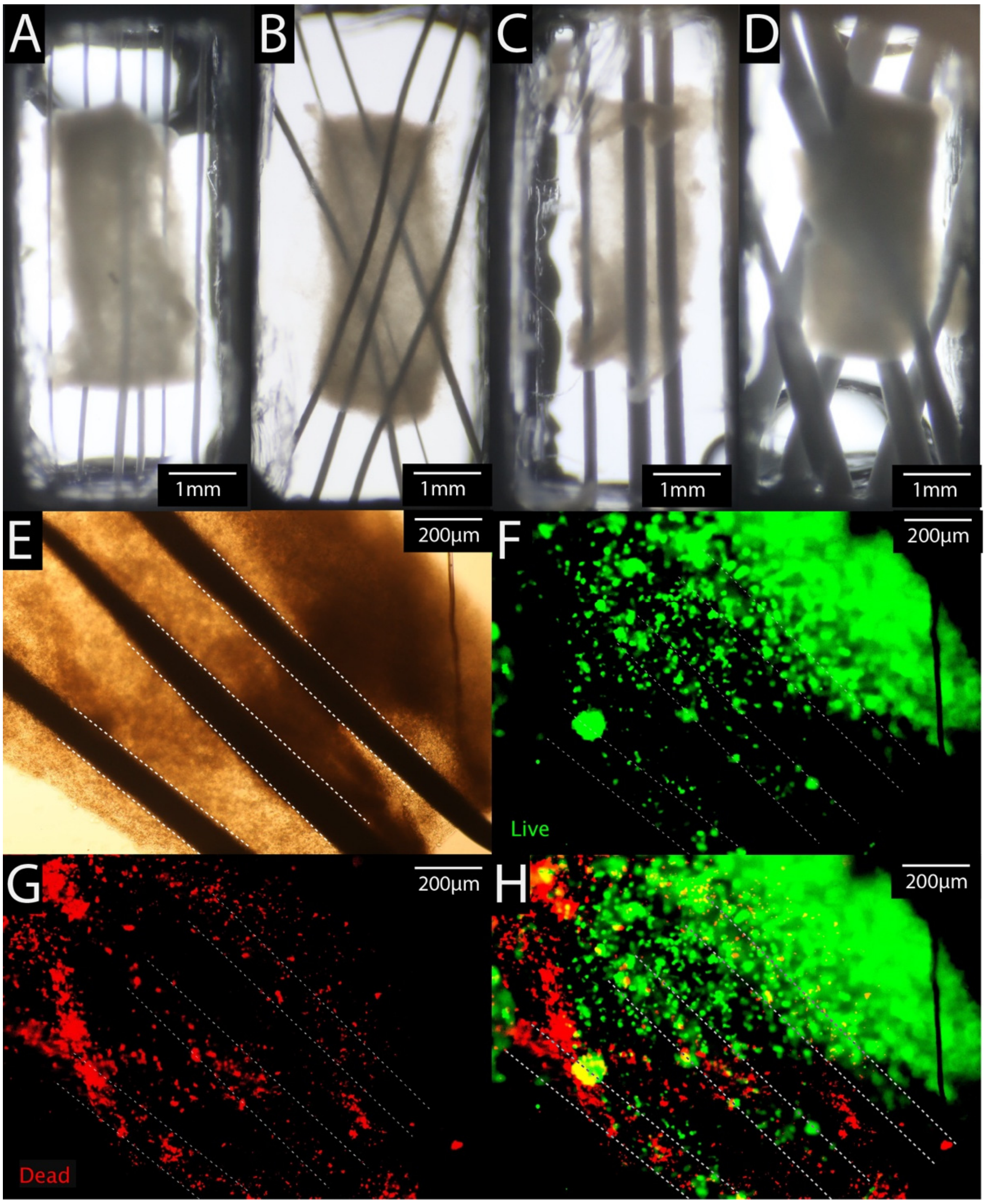


**Supplementary Figure S1:** hiPSC-CMs form viable engineered tissues on fibrous PCL scaffolds. Compacted tissues on (A) 0° Small Fiber, (B) 30° Small Fiber, (C) 0° Large Fiber, and (D) 30° Large Fiber scaffolds. Wide-field fluorescence images of live/dead viability assay on representative 0° small fiber tissue construct, annotated to show fiber location (white dotted lines): (E) bright-field image, (F) live stain, (G) dead stain, (H) composite overlay.

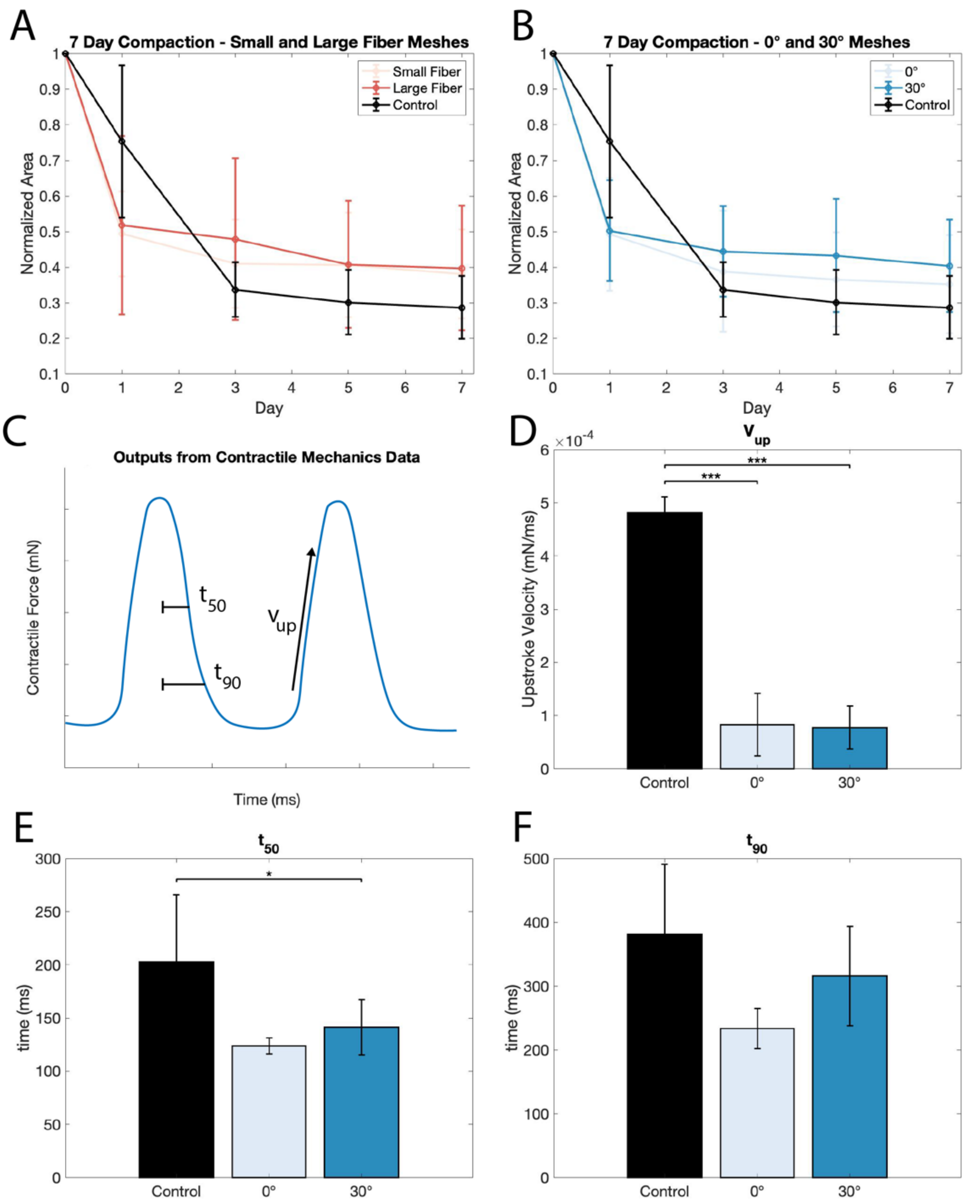


**Supplementary Figure S2**: hiPSC-CMs compact into contractile tissues on fibrous PCL scaffolds. (A) Relative area of tissues on small (SD) and large (LD) fiber scaffolds during days 0-7 of culture, normalized to initial area at day 0 (Small Fiber: 0° and 30° small diameter tissues, n = 6; Large Fiber: 0° and 30° large diameter tissues, n = 27; Control, n = 12). (B) Relative area of tissues on 0° and 30° scaffolds during days 0-7 of culture, normalized to initial area at day 0 (0°: small and large diameter tissues, n = 13; 30°, small and large diameter tissues, n = 20; Control, n = 12). (C) Scheme depicting selected outputs from contractile force data: $t_{50}$, time from peak to 50% relaxation; $t_{90}$, time from peak to 90% relaxation; and $v_{up}$, upstroke velocity, calculated as the rate of force rise from baseline. (D) Upstroke velocity of control, 0°, and 30° tissues. (E) Time to 50% relaxation from peak stress of control, 0°, and 30° tissues. (F) Time to 90% relaxation from peak stress of control, 0°, and 30° tissues. (*, **, and *** indicate $p \leq 0.05$, 0.01, and 0.001, respectively)
